# Polymyxin B Induced Acute Diaphragmatic Paralysis: A Case Report Based on Therapeutic Drug Monitoring

**DOI:** 10.1002/prp2.70196

**Published:** 2025-11-17

**Authors:** Yunli Zhang, Xiaogang Tang, Yan Li, Xia Su, Xiaohong Zhong, Zhai Huang

**Affiliations:** ^1^ Department of Intensive Care Unit, Guangxi Academy of Medical Sciences The People's Hospital of Guangxi Zhuang Autonomous Region Nanning China

**Keywords:** nephrotoxicity, neurotoxicity, polymyxin B, respiratory failure

## Abstract

Polymyxin B (PMB), a last‐resort antibiotic for multidrug‐resistant Gram‐negative infections, carries significant neurotoxicity risks that remain underrecognized in clinical practice. Here, we present a case of life‐threatening diaphragmatic paralysis induced by PMB in a patient with extensive neck and mediastinal infections caused by extensively drug‐resistant *Acinetobacter baumannii* . The patient developed acute respiratory failure due to respiratory paralysis, which resolved completely upon PMB discontinuation. Concurrent use of nephrotoxic agents may have contributed to renal impairment during treatment. This case is helpful in detecting the serious neurotoxic reactions caused by PMB at an early stage; systematic therapeutic drug monitoring combined with real‐time renal function assessment aided in the early detection of toxicity, thereby preventing a potentially fatal outcome.

## Abstract 2

For high‐risk patients (e.g., diabetes, hypoalbuminemia, concurrent sedation/analgesia, or nephrotoxic co‐medications), comprehensive drug interaction evaluation should be routinely implemented. Therapeutic drug monitoring coupled with renal function assessment may serve as a critical strategy to optimize antimicrobial efficacy while minimizing toxicity.

## 1 Introduction

In recent years, infections caused by multidrug‐resistant (MDR) Gram‐negative bacteria in intensive care units (ICUs) have become increasingly severe. In response to this challenge, polymyxin B has re‐emerged as a last‐line therapeutic option against MDR Gram‐negative bacterial infections such as *Pseudomonas aeruginosa* , *Acinetobacter baumannii* , and *Klebsiella pneumoniae* , with its clinical usage showing a significant upward trend [1]. Consequently, the associated drug toxicity profile has attracted increasing clinical attention. Reports of polymyxin‐induced neurotoxicity were primarily documented in the 1960s [2]; however, as polymyxin was gradually replaced by other antibiotics offering better efficacy and lower toxicity (e.g., β‐lactams, cephalosporins, carbapenems), the frequency of such reports declined significantly [3, 4]. Early studies mainly described mild neurological symptoms, including perioral paresthesia or numbness, limb tingling, pruritus, dizziness, and dysarthria [5, 6], while more severe manifestations, such as diaphragmatic dysfunction and life‐threatening respiratory paralysis, were rarely reported. Polymyxin B‐induced severe neurotoxicity remains an underrecognized yet potentially fatal complication. Such adverse events not only prolong ICU stays and increase healthcare expenditures but also pose serious threats to the lives of critically ill patients. Therefore, there is an urgent need to enhance understanding of this rare but severe neurotoxicity and to develop reliable strategies for managing high‐risk patients.

## 2 Case Presentation

**FIGURE 1:**
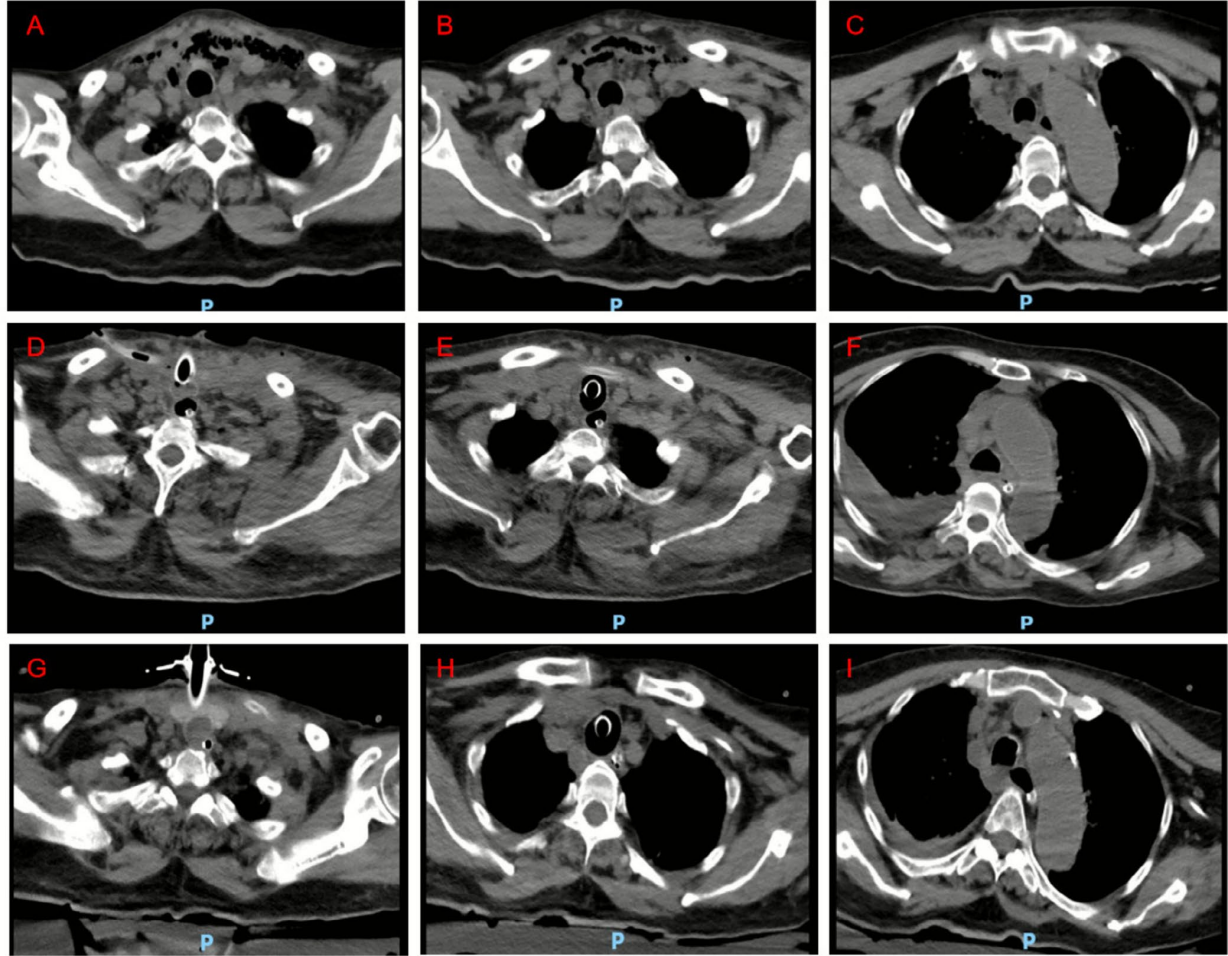
CT images acquired at different time points during the patient's hospitalization. (A–C) April 21; (D–F) May 13; (G–I) June 2.

A 63‐year‐old female patient had poorly controlled hypertension and type 2 diabetes mellitus for more than 10 years. Informed consent of the patient was obtained. The patient presented with persistent pharyngeal pain and cervical swelling lasting 4 days, which progressed to respiratory distress requiring hospital admission on April 21, 2025. The initial cervical computed tomography (CT) scan revealed extensive soft tissue edema with gas formation, extending from the left parapharyngeal space to the anterior mediastinum. Significant thickening was also observed in the left oropharyngeal wall, epiglottis, and laryngopharyngeal folds (Figure 1A–C), indicative of severe necrotizing infection. Although the patient was hemodynamically stable on admission, the presence of immunocompromised status and elevated inflammatory markers (procalcitonin 3.58 ng/mL) prompted empirical antibiotic therapy with piperacillin‐tazobactam. Emergency surgical debridement via a lateral cervical approach and tracheostomy were performed. On April 23, antimicrobial therapy was escalated to meropenem (1 g every 8 h). The patient subsequently developed persistent fever up to 39°C. In the context of a significantly elevated meropenem trough concentration and in the absence of identified pathogens at that time—thus managed empirically for susceptible organisms, the meropenem dose was reduced to 0.5 g every 8 h on April 29. Vancomycin (1 g every 12 h) was added concomitantly for gram‐positive coverage, after which the patient's condition temporarily stabilized. Following temporary clinical stabilization, the patient was transferred to the intensive care unit on May 10 due to persistent high‐grade fever (ranging between 39.8°C and 41.5°C), hypotension (blood pressure: 100/54 mmHg), and progressive respiratory deterioration. Daily output of approximately 100–150 mL of foul‐smelling purulent drainage from two cervical drains necessitated frequent wound dressing changes. Microbiological cultures of wound secretions and tracheal aspirates obtained on May 12 identified extensively drug‐resistant *Acinetobacter baumannii* , resistant to all tested antibiotics including ceftriaxone, ciprofloxacin, gentamicin, ceftazidime, and meropenem, except for colistin and tigecycline. Consequently, for a 60‐kg adult woman with normal liver and kidney function, treatment was initiated with polymyxin B (750,000 IU every 12 h) and tigecycline (50 mg every 12 h), while meropenem was discontinued. Concomitant therapies included therapeutic‐dose heparin, vancomycin (0.5 g q12h), esomeprazole, and intermittent ibuprofen administration (detailed information is shown in Table 1).

**TABLE 1:** Laboratory results during hospitalization.

Date	White blood cell (×10^9^/L)	Neutrophil ratio (%)	Hemoglobin (g/L)	Platelet (×10^9^/L)	Procalcitonin (ng/mL)	Urea (mmol/L)	Creatinine (umol/L)	Uricacid (umol/L)	Potassium (mmol/L)	Trough Concentration (ug/mL)	Intervention
Normal range	3.5–9.5	40–75	130–175	125–350	< 0.5	3.6–9.5	59–104	208–428	3.5–4.5	Vancomycin (10–20) Polymyxin B (2–4)	
2025/4/21	20.14	92.0	111	355	3.58	9.20	56	263	4.05	—	Surgical procedure combined with initial antibiotic therapy (piperacillin‐tazobactam 4.5 g q8h)
2025/4/23	10.07	83.2	104	288	11.15	14.43	107	408	4.40	—	The high fever persisted, antibiotic therapy was escalated to meropenem 1 g q8h
2025/4/29	21.88	85.5	97	456	0.76	7.52	55	181	3.60	Meropenem (22.38)	Persistent high fever did not improve, and coccal infection was considered. The meropenem dose was adjusted to 0.5 g q8h, and vancomycin 1 g q12h was added
2025/5/02	13.18	79.1	104	748	—	7.69	59	152	4.36	Vancomycin (19.02)	
2025/5/05	14.46	80.4	93	792	—	4.94	59	112	4.20	—	
2025/5/07	12.20	78.4	90	815	—	4.37	56	115	5.48	—	
2025/5/09	10.90	79.0	88	740	—	3.81	55	101	4.35	Vancomycin (28.46)	
2025/5/10	12.05	87.6	70	560	0.15	5.60	44	102	4.30	—	Transfer to the ICU
2025/5/12	14.61	77.0	69	483	0.35	5.50	59	132	4.40	—	Multidrug‐resistant *Acinetobacter baumannii* was identified in wound secretions and bronchoalveolar lavage fluid. Meropenem was discontinued, intravenous polymyxin B (75 mg q12 h) was initiated, and the vancomycin dose was reduced to 0.5 g q12 h. Due to abundant purulent drainage, tigecycline (50 mg q12 h) was added to the regimen
2025/5/15	11.12	76.5	60	343	—	9.80	100	186	4.30	Polymyxin B (3.8)	Thoracoscopic and cervical debridement were performed
2025/5/16	10.15	77.9	66	267	3.58	12.00	143	219	3.10	—	
2025/5/17	17.02	92.9	82	270	1.87	14.10	156	244	3.40	Vancomycin (78.05)	Vancomycin was discontinued. An attempted weaning from mechanical ventilation failed, prompting evaluation for suspected neuromuscular pathology
2025/5/18	18.35	94.0	98	316	2.25	19.30	192	295	4.40	—	
2025/5/19	14.07	81.3	93	314	—	21.30	193	297	3.70	Polymyxin B (5.1) Vancomycin (31.03)	
2025/5/22	10.54	73.6	89	326	—	24.90	238	221	3.66	—	
2025/5/23	10.40	70.5	80	302	—	30.80	297	226	4.10	Polymyxin B (6.82)Vancomycin (17.21) Tigecycline (0.46)	Considering the potential neuromuscular toxicity associated with polymyxin B, which may lead to diaphragmatic dysfunction, polymyxin B was discontinued. The antibiotic therapy was adjusted to meropenem 0.5 g q8h
2025/5/24	10.14	72.7	78	298	—	32.80	324	263	4.90	—	
2025/5/26	12.69	92.2	85	362	—	37.40	396	291	5.10	Polymyxin B (1.16)	The patient was successfully weaned from mechanical ventilation and extubated. Vancomycin was discontinued due to a significant decline in renal function
2025/5/28	18.83	75.3	85	493	—	48.80	491	385	6.03	—	Transferred to specialty ward after condition improved
2025/5/29	14.48	67.1	82	456	—	54.45	505	407	5.50	—	
2025/6/02	12.93	61.4	85	505	—	47.62	380	388	4.01	—	
2025/6/07	—	—	—	—	—	21.39	152	235	3.50	—	Tigecycline discontinued due to controlled infection and rapidly improving renal function
2025/6/10	—	—	—	—	—	—	—	—	—	—	Discharge from hospital

A repeat CT scan on May 13 revealed disease progression with the presence of a new loculated pleural effusion (Figure 1D–F), necessitating radical debridement of deep neck structures and thoracoscopic drainage of pleural pus on May 19. Postoperative patient‐controlled intravenous analgesia was administered with the following formulation: oliceridine fumarate injection 30 mg + nalbuphine hydrochloride injection 60 mg + tropisetron injection 10 mg, diluted with 0.9% normal saline to a total volume of 200 mL, and delivered at a pump rate of 4 mL/h. On May 17, following partial relief of respiratory distress and fever, we attempted ventilator weaning, which precipitated acute respiratory failure (PaCO_2_ 110 mmHg) and encephalopathy, accompanied by markedly reduced respiratory movement despite preserved limb strength and sensory function.

Initially, the observed diaphragmatic dysfunction was suspected to be secondary to iatrogenic phrenic nerve injury during cervical abscess debridement or thoracoscopic surgery. However, upon review of surgical records, it was confirmed that dissection was limited to the superficial cervical musculature without extension into deeper planes such as the carotid sheath, thereby making direct nerve injury unlikely. A second differential consideration included neuromuscular disorders such as acute inflammatory demyelinating polyneuropathy or myasthenia gravis. Further blood tests demonstrated a significantly decreased serum cholinesterase level (1568 U/L; normal range: 5000–12 000 U/L), while creatine kinase levels remained within normal limits (13 U/L; normal range: 4–200 U/L). The neostigmine test was negative, and anti‐acetylcholine receptor antibodies were undetectable, leading to the exclusion of myositis and myasthenia gravis. We also used the MRC comprehensive scoring tool to assess the possibility of acquired weakness in the intensive care unit. A score of 60 (a score below 48 indicates the presence of ICU‐AW), and clinical exclusion of ICU‐AW was carried out.

Subsequently, drug‐induced respiratory paralysis was considered. At this time, the trough vancomycin level was elevated at 78.05 μg/mL (therapeutic range: 10‐20 μg/mL), in conjunction with ongoing PCA use, raising suspicion for pharmacologically induced neuromuscular impairment. Consequently, both vancomycin and PCA were promptly discontinued. By May 23, the vancomycin level had normalized to 17.21 μg/mL; however, the patient remained ventilator‐dependent. Meanwhile, in parallel with the progressive exacerbation of acute kidney injury, we found that the serum concentration of polymyxin B was 6.82 μg/mL (target: 2–4 μg/mL), which reached supratherapeutic levels (Detailed laboratory results are provided in Table 1). The Naranjo adverse drug reaction probability scale of the patient was 7 (Table 2), which showed a probable relationship between PMB infusion and respiratory failure. Therefore, polymyxin‐induced neuromuscular blockade was suspected, and PMB was discontinued immediately. Three days later, its serum concentration declined to 1.16 μg/mL, allowing successful ventilator liberation.

**TABLE 2:** Scores obtained for the patient based on the Naranjo Adverse Drug Reaction Probability Scale.

Naranjo adverse drug reaction probability scale	
Questions	Score
Are there previous conclusive reports of this reaction?	1
Did the adverse event appear after the drug was given?	2
Did the adverse reaction improve when the drug was discontinued, or a specific antagonist was given?	1
Did the adverse reaction reappear on readministering the drug?	0
Were there other possible causes for the reaction?	0
Did the adverse reaction reappear on administration of placebo?	0
Was the drug detected in the blood or other fluids in toxic concentrations?	1
Was the reaction worsened on increasing the dose? Or, was the reaction lessened on decreasing the dose?	1
Did the patient have a similar reaction to the drug or a related agent in the past?	0
Was the adverse event confirmed by any other objective evidence?	1
Total score	7

*Note:* ≥ 9, Definite; 5–8, Probable; 1–4, Possible; ≤ 0, Doubtful.

The patient was transferred to the general ward on May 28 and exhibited rapid recovery of renal function thereafter. Serial imaging follow‐up on June 2 demonstrated substantial resolution of both cervical infection and empyema (Figure 1G–I). Her clinical condition continued to improve steadily, culminating in successful hospital discharge on June 10.

## 3 Discussion

We present a case of severe respiratory paralysis characterized by progressive diaphragmatic weakness, restricted thoracic excursion, and hypercapnic respiratory failure that developed in a patient following 1 week of PMB therapy. Notably, the patient exhibited no clinical manifestations of tetany, arrhythmias, seizures, or involuntary movements, and electrolyte levels remained within normal limits throughout the treatment course. Potential alternative etiologies were systematically ruled out. Laboratory analysis revealed a supratherapeutic PMB serum concentration of 6.82 μg/mL. Following discontinuation of PMB, the serum level decreased to within the safety range within 72 h, coinciding with the complete resolution of the respiratory paralysis.

Emerging evidence suggests that the incidence of PMB‐associated neurotoxicity has declined to approximately 3%–7%, likely attributable to optimized dosing strategies and enhanced therapeutic drug monitoring [3, 4]. While most neurotoxic effects are mild in nature, respiratory paralysis remains a rare yet potentially life‐threatening complication associated with PMB use [7, 8]. Ning et al. [9] described a case of multidrug‐resistant *Pseudomonas aeruginosa* infection following spinal disc surgery, in which conventional‐dose colistin therapy resulted in progressive limb weakness and frequent falls after 2 weeks, progressing to dysarthria and respiratory failure by the fifth week. Our patient developed isolated respiratory distress after only 1 week of PMB therapy, without sensory deficits or altered mental status. This case underscores the clinical heterogeneity of polymyxin‐induced neurotoxicity and highlights the importance of early recognition of respiratory paralysis by clinicians, particularly in critically ill patients with aphasic, comatose, or under sedation, who are unable to voluntarily report or precisely characterize their symptoms.

Several case reports have documented that colistin‐induced neurotoxicity is typically reversible, with the underlying mechanism possibly involving a presynaptic neuromuscular blockade [3]. In our case, the absence of anti‐acetylcholine receptor antibodies and the complete recovery following drug withdrawal support this hypothesis. The neuromuscular blockade induced by polymyxins appears to be noncompetitive in nature and not amenable to reversal by neostigmine, resembling the pathophysiology of organophosphate poisoning. Organophosphorus compounds inhibit acetylcholinesterase activity, leading to excessive accumulation of acetylcholine at the neuromuscular junction, resulting initially in hyperexcitability followed by depolarizing blockade, clinically manifesting as diaphragmatic weakness. This phenomenon mirrors cases of congenital cholinesterase deficiency, which similarly presents with impaired diaphragmatic function, emphasizing the critical role of this enzyme in neuromuscular transmission. In the current case, persistently reduced serum cholinesterase levels were observed, suggesting that pre‐existing cholinesterase insufficiency may have predisposed the patient to an increased susceptibility to polymyxin‐induced neuromuscular blockade.

Regarding renal effects, the patient had normal baseline renal function at admission. However, after 5 days of combined PMB and vancomycin therapy, serum creatinine rose rapidly to 297 μmol/L. At that time, vancomycin trough levels were elevated (78.05 μg/mL). Cessation of both agents led to gradual renal recovery. Notably, when vancomycin was later reintroduced as monotherapy, no nephrotoxicity was observed. This temporal pattern suggests PMB played a primary role in the initial renal injury.

Known risk factors for PMB nephrotoxicity include advanced age, diabetes, hypoalbuminemia, and concomitant nephrotoxic drugs [10, 11]. Combination therapy with vancomycin increases acute kidney injury risk by more than twofold, likely due to synergistic toxicity [12]. Such interactions can prolong exposure to toxic drug levels [13], as seen in our patient who developed toxicity despite standard PMB dosing. These observations reinforce the narrow therapeutic index of PMB and emphasize the importance of combining therapeutic drug monitoring with creatinine clearance–based dosing to balance efficacy and safety.

This case reports a serious adverse event suspected to be caused by the neurotoxic effect of polymyxin B, emphasizing the necessity of clinical vigilance, especially in high‐risk patients (such as those with diabetes, hypoalbuminemia, or those receiving sedation treatment). Taking proactive drug monitoring measures combined with renal function assessment may be a better approach to optimize treatment in such situations. However, it is important to acknowledge the limitations inherent in a single case report, including the inability to establish causality and the potential influence of unmeasured confounders such as critical illness itself and concomitant medications. Therefore, the findings and management strategies suggested here should be interpreted with caution and require further validation in larger prospective studies.

## Author Contributions

**Zhai Huang:** conceptualization, review and editing. **Yunli Zhang:** data curation, review and editing; writing – original draft. **Xiaogang Tang:** data curation; writing – original draft. **Yan Li:** investigation. **Xia Su:** visualization. **Xiaohong Zhong:** supervision.

## Disclosure

PI Statement: The authors confirm that the project leader for this paper is Yunli Zhang, who had direct clinical responsibility for the patient.

## Ethics Statement

The publication of this case report complies with the Declaration of Helsinki. The Ethics Committee of Guangxi Zhuang Autonomous Region People's Hospital waived the requirement for approval as only anonymized data were used. Patient consent was obtained for publication.

## Consent

Written informed consent was obtained from the patient for publication of this case report and any accompanying images. The consent form is retained by the authors and is available for review by the Editor if requested.

## Conflicts of Interest

The authors declare no conflicts of interest.

## Data Availability

The data that support the findings of this study are available on request from the corresponding author. The data are not publicly available due to privacy or ethical restrictions.
